# Prevalence and short‐term changes of cognitive dysfunction in young ischaemic stroke patients

**DOI:** 10.1111/ene.13879

**Published:** 2019-01-09

**Authors:** D. Pinter, C. Enzinger, T. Gattringer, S. Eppinger, K. Niederkorn, S. Horner, S. Fandler, M. Kneihsl, K. Krenn, G. Bachmaier, F. Fazekas

**Affiliations:** ^1^ Department of Neurology Medical University of Graz Graz Austria; ^2^ Division of Neuroradiology, Vascular and Interventional Neuroradiology Medical University of Graz Graz Austria; ^3^ Institute for Medical Informatics, Statistics and Documentation Medical University of Graz Graz Austria

**Keywords:** acute, changes, cognition, processing speed, stroke, young

## Abstract

**Background and purpose:**

Information on the prevalence and course of post‐stroke cognitive impairment in young stroke patients is limited. The aim was to assess a consecutive sample of acute young ischaemic stroke patients (18–55 years) for the presence and development of neuropsychological deficits.

**Methods:**

Patients prospectively underwent a comprehensive clinical and cognitive assessment, examining general cognitive function, processing speed, attention, flexibility/executive function and word fluency within the first 3 weeks after hospital admission (median assessment at day 6) and at a 3 months’ follow‐up (FU). Cognitive dysfunction was defined in comparison to age‐standardized published norms.

**Results:**

At baseline (*N* = 114), deficits were highly prevalent in processing speed (56.0%), flexibility/executive function (49.5%), attention (46.4%) and general cognitive function (42.1%). These frequencies were comparable for those with FU assessment (*N* = 87). In most domains, cognitive performance improved within 3 months, except for word fluency. However, in about one‐third of patients, cognitive deficits (as defined by 1.5 standard deviations below the standardized mean) were still present 3 months after stroke. At FU, 44.0% were impaired in the domain flexibility/executive function, 35.0% in processing speed and 30.0% in attention.

**Conclusions:**

The high prevalence of cognitive deficits in acute young patients with ischaemic stroke highlights the importance of early post‐stroke cognitive assessment to capture a patient's dysfunction in a comprehensive manner and to offer adequate rehabilitation. The role of factors which promote neuropsychological deficits needs further exploration.

## Introduction

The incidence of stroke in young adults (aged 18–55 years) is increasing 1, 2, 3 and about 10% of all strokes occur in ‘young’ adults below 50 years of age 4, 5, rising to 18.6% if the upper age limit is set at 55 years 1. These patients suffer from serious physical, cognitive and psychosocial consequences and the prognosis of young patients with stroke does not appear as favorable as previously thought 4, 6.

In contrast to a fairly good motor recovery, the few studies assessing post‐stroke cognition in young stroke patients report alarmingly high prevalence rates of cognitive impairment 7, 8. Cognitive impairment in up to 60% has been reported 4–12 months post‐stroke 9. Even after 11 years 8, 10 cognitive impairment was still present in about 35% of patients, affecting activities of daily living, quality of life and return to work independently of physical recovery 7, 11. None of the above mentioned studies explored the course of cognitive impairment by assessing the same cohort twice which precludes information on the dynamics of post‐stroke cognitive function and thus may lead to an overestimation or underestimation of the problem due to improvement or deterioration over time. A recent study, investigating patients from 18 to 65 years, showed that cognitive function mostly improves within the first 6 months post‐stroke, with only little cognitive recovery thereafter, highlighting the fact that changes occur mostly early on 12.

Although it has been shown that early neuropsychological information provides valid information to predict post‐stroke functional outcome 13, in acute stroke usually short cognitive screening tests rather than a comprehensive neuropsychological test battery are used, although the precision of these instruments has been questioned 12. To our knowledge, so far only one study 14, investigating 20 patients with infratentorial infarcts, assessed cognitive function in acute young stroke patients. Those authors highlight the value of early post‐stroke cognitive assessment, given observed associations of acute cognitive function with working capacity after 12 months, but unfortunately did not report if or to what degree cognitive function changed at follow‐up.

In a prospective single‐center study, the aim was therefore to assess the prevalence and course of cognitive dysfunction in a consecutive sample of acute young stroke patients by detailed neuropsychological assessment.

## Methods

### Patients

From February 2016 to April 2018, all consecutive patients aged 18–55 years with an acute imaging‐proven stroke were invited to participate in our Stroke in the Young Study (*N* = 214). Within this period, 64 patients were not able or did not want to participate in our study. In all, 150 young stroke patients, comprising 114 (76%) patients suffering from ischaemic stroke who were the target group for this analysis, were thus examined. Recruitment details including a flowchart (Fig. S1) are provided in Data S1.

The study was approved by the ethics committee of the Medical University of Graz. All participants gave written informed consent.

### Clinical assessment

All patients underwent routine neurological examination and an extensive neuropsychological assessment during the initial stay at our department because of the acute event and at a pre‐specified 3 month follow‐up.

During clinical routine work‐up, all patients received brain magnetic resonance imaging (MRI) except for three patients undergoing only computed tomography (CT). Neurological symptoms, vascular risk factors, stroke severity [National Institutes of Health Stroke Scale (NIHSS); modified Rankin Scale (mRS)], lesion side and vascular territory were assessed for every patient. Stroke etiology was classified according to TOAST 15.

### Neuropsychological assessment

The neuropsychological test battery included four tests: the Montreal Cognitive Assessment (MoCA), the Symbol Digit Modalities Test (SDMT) and the Comprehensive Trail Making Test (CTMT) (subtests 2 and 5). The time difference between the two subtests, which is considered to specifically reflect executive function, independently of motor speed and visual scanning speed, was also assessed 16. Two subtests of a word fluency test [Regensburger Wortflüssigkeitstest (RWT)] were additionally applied. All tests were evaluated using standardized published age‐related norms. Depression and anxiety were assessed with the Hospital Anxiety and Depression Scale (HADS). The neuropsychological assessment took about 30 min. In line with prior studies, clinical impairment was defined by 1.5 standard deviations below the standardized mean 8, 17.

Details of the neuropsychological assessment and information on the statistical analysis are available in Data S1.

## Results

### Patient characteristics

Demographics, clinical and neuroimaging characteristics of the investigated patients with ischaemic stroke are presented in Table 1.

**Table 1:** Demographics, clinical and MRI characteristics of patients at baseline, for the entire cohort (*N* = 114) and for those patients with available FU (*N* = 87)

Baseline characteristics	*N* = 114	*N* = 87
Age at baseline (years, SD)	44.5 (9.5)	43.8 (9.8)
Sex, female *N* (%)	41 (36%)	31 (35.6%)
Education, years, median (IQR)	12 (3)	12 (3)
Clinical characteristics, median (IQR)		
NIHSS	2 (3)	2 (3)
mRS	1 (2)	1 (2)
Time to neuropsychological assessment, days	6 (5)	6 (4)
Duration hospital stay, days	10 (11)	10 (8)
Clinical symptoms, *N* (%)		
Hemiparesis	48 (42.1)	37 (42.5)
Facial paralysis	39 (34.2)	28 (23.2)
Hemisensory symptoms	49 (43.0)	34 (39.1)
Dysarthria	28 (24.6)	21 (24.1)
Visual impairment	16 (14.0)	16 (18.4)
Aphasia	22 (19.3)	16 (18.4)
Vascular risk factors, *N* (%)		
Hypertension	49 (43.0)	50 (57.5)
Atrial fibrillation	7 (6.1)	7 (8.0)
Hyperlipidemia	58 (50.9)	44 (50.6)
Diabetes	12 (10.5)	7 (8.0)
Smoking	53 (46.5)	41 (47.1)
History of prior stroke	12 (10.5)	7 (8.0)
Vascular territory, *N* (%)		
Anterior cerebral artery	1 (0.9)	1 (1.1)
Middle cerebral artery	51 (44.7)	34 (39.1)
Posterior cerebral artery	11 (9.6)	8 (9.2)
Vertebrobasilar arteries	35 (30.7)	31 (35.6)
Multiple territories	16 (14.1)	13 (14.9)
Etiology by TOAST, *N* (%)		
Large‐artery atherosclerosis	14 (12.3)	12 (13.7)
Cardioembolism	18 (15.8)	10 (11.4)
Small‐artery occlusion	24 (21.1)	16 (18.4)
Other determined	9 (7.8)	7 (8.0)
Undetermined	49 (43.0)	42 (48.3)
Side of supratentorial lesion, *N* (%)	N = 70	N = 49
Right side	30 (42.9)	21 (42.9)
Left side	33 (47.1)	24 (48.9)
Bilateral	7 (10.0)	4 (8.2)

FU, follow‐up; IQR, interquartile range; MRI, magnetic resonance imaging; mRS, modified Rankin Score; NIHSS, National Institutes of Health Stroke Scale.

The mean age of our patient sample was 44.5 years (SD 9.5) at stroke onset and 36% were female. Median stroke severity was mild (median NIHSS = 2, median mRS = 1). About half of the patients had a middle cerebral artery infarct (51%); 39% had a lesion in the right side and 45% in the left side of the brain. The majority were classified as a stroke of undetermined cause (43%). Further details of patient characteristics including clinical symptoms and vascular risk factors are presented in Table 1. Prevalence rates of clinical deficits at FU are given in the Data S1 and Table S1.

### Prevalence of cognitive impairment

Within the first 3 weeks after hospital admission (median assessment at day 6) more than 40% of patients showed impairment in general cognitive function and attention (Table 2). Impairment in the domains processing speed and flexibility/executive function was even more frequent. Lower percentages of impairment (30%–20%) were observed for the word fluency test. However, 14 patients were not able to perform the word fluency test at all. Depression or anxiety scores of ≥11 (representing moderate to severe impairment) were observed in a fifth of patients (Table 2).

**Table 2:** Prevalence of patients with cognitive impairment at baseline (1.5 standard deviations below standardized mean)

Assessment	BL impaired *N* (%) *N* = 114^a^	BL impaired *N* (%) *N* = 87
MoCA: global cognition (<26 raw score)	48 (42.1)	36 (41.4)
SDMT: processing speed	61 (56.0)	48 (55.2)
CTMT‐2: attention	52 (46.4)	39 (45.3)
CTMT‐5: flexibility	55 (49.5)	42 (49.4)
RWT: phonological word fluency	31 (30.1)	23 (28.8)
RWT: semantic word fluency	20 (20.0)	13 (16.3)
HADS: Depression	22 (20.0)	16 (20.0)
HADS: Anxiety	21 (19.1)	16 (20.0)

CTMT, Comprehensive Trail Making Test; HADS, Hospital Anxiety and Depression Scale; MoCA, Montreal Cognitive Assessment; RWT, Regensburger Wortflüssigkeitstest; SDMT, Symbol Digit Modalities Test.

a At baseline (BL), scores of the cognitive subtests were available for *N* = 109 (SDMT), *N* = 112 (CTMT‐2), *N* = 111 (CTMT‐5), *N* = 100 (RWT), *N* = 110 (HADS).

### Course of cognitive impairment

**Figure 1:**
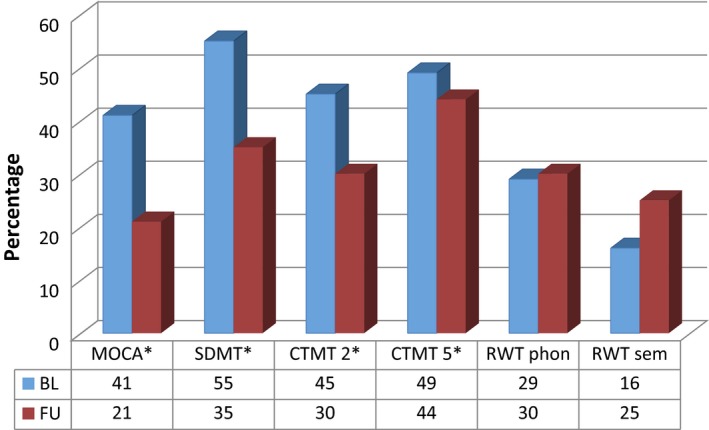
Prevalence changes of patients with cognitive impairment (1.5 standard deviations below standardized mean). *P <* *0.001. At FU, scores of the cognitive subtests were available for *N* = 86 (CTMT‐2), *N* = 85 (CTMT‐5) and *N* = 80 (RWT, HADS). [Color figure can be viewed at wileyonlinelibrary.com]

Neuropsychological follow‐up assessment 3 months post‐stroke was available for 87 patients, with demographics, clinical and MRI characteristics comparable to the baseline cohort (Table 1). Prevalence rates of cognitive impairment were also comparable between patients with and without FU assessment (Table 2). At FU patients had improved in general cognitive ability, processing speed, attention, flexibility and executive function (Table 3). However, cognitive deficits were still present 3 months after stroke in about one‐third of them. No changes were observed in phonological and semantic word fluency (Table 3). The change in the rates of abnormal test performance from baseline to 3 months’ FU is shown in Fig. 1. Rates of depression and anxiety were significantly lower at FU and patients had also improved clinically regarding the NIHSS and mRS scores (Table 3).

**Table 3:** Evolution of cognitive test performance, anxiety, mood and clinical scores from baseline to FU (*N* = 87)

Assessment	BL	FU	*P*
Days to FU assessment		99.0 (45.0)	
MoCA	26.0 (5.0)	27.0 (3.0)	<0.001
SDMT	−1.5 (1.4)	−0.9 (2.0)	<0.001
CTMT‐2: *T* score	37.0 (15.0)	40.0 (14.0)	<0.001
CTMT‐5: *T* score	36.0 (20.0)	39.0 (15.0)	<0.001
CTMT‐5 – CMT‐2 in seconds	29.0 (45.0)	23.0 (32.0)	0.021
RWT: phonological	15.0 (35.0)	12.8 (20.0)	0.154
RWT: semantic	27.5 (38.4)	29.0 (41.0)	0.811
HADS: Depression	3 (4)	2 (3)	0.007
HADS: Anxiety	6 (7)	2 (4)	0.031
NIHSS	2 (3)	0 (1)	<0.001
mRS	1 (2)	1 (0)	0.003

BL, baseline; CTMT, Comprehensive Trail Making Test; HADS, Hospital Anxiety and Depression Scale; MoCA, Montreal Cognitive Assessment; mRS, modified Rankin Score; NIHSS, National Institutes of Health Stroke Scale; RWT, Regensburger Wortflüssigkeitstest; SDMT, Symbol Digit Modalities Test; *T* score, T normative score.

Scores are presented as median (IQR).

## Discussion

This prospective FU study indicates that cognitive impairment is highly prevalent in young patients with acute ischaemic stroke and still affects a third of them after 3 months. In line with prior studies, processing speed, assessed by the SDMT, was most severely impaired at baseline 8, 10, 12, 14, 17. Furthermore, flexibility/executive function was impaired in almost half of young patients at baseline and still in 40% 3 months after stroke. These two domains have been closely associated with functional recovery, return to work and the quality of life of stroke patients 11, 17, 18, 19, 20 and therefore should be examined in clinical routine to recognize existing deficits particularly also in young patients with stroke.

Surprisingly there is still limited awareness and recognition of cognitive deficits in young stroke patients and data about the course of cognitive recovery in young stroke with regard to short‐ and long‐term changes is scarce. Not unexpectedly the strongest progress in post‐stroke recovery appears to occur within the first days and weeks 21, and our FU assessment after 3 months was therefore planned to capture these short‐term changes. Although significant improvements were noted across almost all cognitive domains about a third of patients still suffered from cognitive impairment after 3 months. To what extent cognitive recovery continues beyond this time period will need further studies. In this regard, important insights are expected from the ongoing Observational Dutch Young Symptomatic Stroke Study (ODYSSEY), which aims to include 1500 patients and will be assessing cognitive function 6 weeks and 1 year after the index event 22. A recent study suggests that most cognitive recovery happens within the first 6 months, with little change thereafter 12, highlighting the importance of exploring the dynamic course of post‐stroke cognitive impairment especially early on.

Testing of neuropsychological functions early on is also important to inform post‐stroke rehabilitation strategies. In line with prior studies 7, 8, our patients showed relatively few physical deficits at baseline with good recovery after 3 months. Therefore the necessity of neurorehabilitation frequently focusing predominantly on motor functions is often not seen. Whilst post‐stroke cognitive impairment may be even more disabling than physical dysfunction such deficits may be easily overseen and need to be specifically looked for in order to provide adequate rehabilitation including specific cognitive training. To identify specific targets for cognitive rehabilitation, a short neuropsychological assessment including a global test and tests such as the SDMT and CTMT appears cost efficient. Our assessment lasted for 30 min, was feasible in the majority of patients and captured a high rate of cognitive dysfunction. Previous studies have already highlighted that an early cognitive assessment within the first weeks post‐stroke provides valuable information on long‐term cognitive performance, return to work and functional outcome 7, 11, 13, 23.

Consistent with other studies, hypertension, smoking and hyperlipidemia were the most common vascular risk factors in young stroke patients 24, 25. Interestingly, Lu *et al*. 25 reported that vascular risk factors (e.g. hypertension, smoking, diabetes) aggravate cognitive dysfunction in young ischaemic stroke patients. To further substantiate and clarify this association it would be necessary to correct for confounding factors such as age and coexisting morphological damage as it is known from healthy subjects that adverse effects of vascular risk factors on cognition are mediated by covert vascular brain injury such as white matter hyperintensities, covert infarcts and lacunes 26, 27, 28, 29, 30. However, as this was not the major emphasis of our study and because of the limited number of patients these aspects could not be adequately examined and larger and more prolonged studies will be needed to understand these complex interactions.

In line with previous studies, no correlation was found between cognitive impairment and vascular territory or lesion side 9, 31. Previous studies report that left hemisphere stroke might lead to more severe cognitive impairment 27, 32, 33, which is frequently explained by a strong focus on language in the cognitive assessment. Our study sample might be too small or heterogeneous to identify possible differences regarding lesion side and vascular territory. As this was an exploratory study lesion size also was not assessed.

Our study has additional limitations. First, sensorimotor impairments may have influenced our prevalence estimates for cognitive impairment, particularly on timed tests. However, in our cohort only minor motor impairment was present and such influences should therefore be small. Secondly, a specific subtest for memory function was not included, given our focus on the most crucial domains in young stroke patients and time constraints for the neuropsychological assessment. Thirdly, information on the presence and severity of white matter hyperintensities, other vascular damage or brain atrophy, which might add important information on the association between vascular risk factors, covert brain injury and cognitive function, was not included. However, compared to older stroke samples, such influences might be less pronounced, given the mean age of 45 years in our sample. Fourthly, our study is not representative of all young stroke patients as patients with hemorrhagic and more severe strokes which could not be tested were excluded. Yet those patients are likely to suffer from cognitive disturbances to an even greater extent. Furthermore, patients attending the FU could be biased towards patients with milder deficits.

In conclusion, cognitive deficits are highly prevalent in young stroke patients in the acute and subacute stage. An early short, comprehensive neuropsychological assessment is feasible and provides important information to offer cognitive rehabilitation. The contributing effects of vascular risk factors and concomitant vascular brain damage on cognitive function need further investigation with an extended FU period.

## Disclosure of conflicts of interest

The authors declare no financial or other conflicts of interest.

## Funding

Daniela Pinter receives funding from the Austrian Science Fund (FWF): T690‐B23.

## Supporting information


**Data S1.** Methods.
**Figure S1.** Flowchart of participants and non‐participants at baseline and follow‐up.
**Table S1.** Prevalence of clinical deficits at the 3 month follow‐up (*N* = 87). Motor and sensory impairment, aphasia and visual impairment according to the NIHSS. Prevalence is indicated as number of patients *N* (%).
