# Supplementary material for: Prevalence and short‐term changes of cognitive dysfunction in young ischaemic stroke patients
Source: Eur J Neurol. 2019 Jan 9;26(5):727–32. doi: 10.1111/ene.13879 (PMC6491967; doi:10.1111/ene.13879)
Supplement: Supplementary file 1 — Data S1. Methods. Figure S1. Flowchart of participants and non‐participants at baseline and follow‐up. Table S1. Prevalence of clinical deficits at the 3 month follow‐up (N = 87). Motor and sensory impairment, aphasia and visual impairment according to the NIHSS. Prevalence is indicated as number of patients N (%). [file ENE-26-727-s001.docx]

**Prevalence and short-term changes of cognitive** **dysfunction** **in young ischemic stroke patients**

Daniela Pinter^1^, Christian Enzinger^1,2^, Thomas Gattringer^1^, Sebastian Eppinger^1^, Kurt Niederkorn^1^, Susanna Horner^1^, Simon Fandler^1^, Markus Kneihsl^1^, Kathrin Krenn^1^, Gerhard Bachmaier^3^ and Franz Fazekas^1^

**Methods**

**Patients**

From February 2016 to April 2018, all consecutive patients with an acute imaging-proven stroke aged 18 to 55 years were invited to participate in our “*Stroke in the Young Study*” (N=214). Within this period, 64 patients were not able or did not want to participate in our study (Figure S1). Eight patients could not be tested due to lacking German skills impeding understanding the informed consent and/or cognitive assessment. For 24 patients an assessment was not feasible because of too severe impairment, e.g. global aphasia, intensive care, re-infarct during hospital stay. 21 patients were transferred to another hospital or discharged before cognitive assessment was feasible. Six patients suffered from serious pre-stroke psychiatric or oncological diseases precluding cognitive assessment and five patients did not consent to participate in our study. We examined 150 young stroke patients, comprising 114 (76%) patients suffering from ischemic strokes, 11 (7.3%) with hemorrhagic stroke, 9 (6.0%) with subarachnoidal bleeding and 16 (10.7%) with a cerebral sinus venous thrombosis (Figure S1). Self-reported smoking was assessed in a semistructured interview during the clinical examination. History of stroke included all main stroke types.

**Details of the neuropsychological assessment**

The neuropsychological test battery included four neuropsychological tests. First, the Montreal Cognitive Assessment (MoCA) [1], a brief screening tool for global cognition comprising several domains (e.g. visuospatial abilities, memory, attention, orientation) was applied. Subsequently, the Symbol Digit Modalities Test (SDMT) was used to assess processing speed, concentration and visuomotor skills [2]. Furthermore, two subtests of the Comprehensive Trail Making Test (CTMT-2 and 5) were applied to assess attention, concentration, and cognitive flexibility, set-shifting or executive function [3]. We also assessed the time difference between part 5 and 2 of the CTMT, which is considered to specifically reflect executive function, independently of motor speed and visual scanning speed [4]. As phonological and semantic fluency tasks involve different cognitive processes and brain regions [5,6], we additionally applied two phonological (Baseline: letter S, change G-R, Follow-Up (FU): letter P, change H-T) and two semantic subtests (Baseline: First names, Clothing-Flowers, FU= Animals, Sports-Fruit) of a word fluency test (“Regensburger Wortflüssigkeitstest”, RWT), [7] each lasting two minutes. Parallel versions for the baseline and FU were used for all assessments except for the CTMT. All these tests were evaluated using standardized published norms and have age-related norms from 18-55 years of age in German language were relevant. In addition, we also assessed depression and anxiety with the German version of the Hospital Anxiety and Depression Scale (HADS) [8].

**Statistical analysis**

Demographic, clinical and neuropsychological scores were analyzed with the Statistical Package of Social Science (IBM SPSS Statistics 23). The level of significance was set at 0.05. The Kolmogorov–Smirnov test assessed normality of data distribution. To assess potential changes between baseline and FU the Wilcoxon test (for non-normally distributed variables) or paired t-test (for normally distributed continuous variables) was applied.

**Results**

**Patient Characteristics**

Prevalence rates of clinical impairment (motor and sensory impairment, aphasia and visual impairment according to the NIHSS) at the three months follow-up (FU) are presented in Table S1.

**References:**

1. Nasreddine ZS, Phillips N, Bédirian V, Charbonneau S, Whitehead V, Collin I, et al. The Montreal Cognitive Assessment, MoCA: a brief screening tool for mild cognitive impairment. *J Am Geriatr Soc* 2005; **53**:695–9.

2. Smith A. Symbol Digit Modalities Test. 13th edition. *Manual.* 2000.

3. Reynolds CR. Comprehensive Trail Making Test: Examiner’s Manual. Austin, Texas: *PRO-ED*; 2002.

4. Arbuthnott K, Frank J. Trail Making Test , Part B as a Measure of Executive Control : Validation Using a Set-Switching Paradigm. *Journal of Clinical and Experimental Neuropsychology* 2010; **22**:37–41.

5. Biesbroek JM, van Zandvoort MJE, Kappelle LJ, Velthuis BK, Biessels GJ, Postma A. Shared and distinct anatomical correlates of semantic and phonemic fluency revealed by lesion-symptom mapping in patients with ischemic stroke. *Brain Struct Func*t 2016; **221**:2123–34.

6. Fishman KN, Ashbaugh AR, Lanctôt KL, Cayley ML, Herrmann N, Murray BJ, et al. Apathy, not depressive symptoms, as a predictor of semantic and phonemic fluency task performance in stroke and transient ischemic attack. *J Clin Exp Neuropsychol* 2017; **1**:1–13.

7. Aschenbrenner S, Tucha O, Lange K. Regensburger Wortflüssigkeitstest (RWT). Hogrefe: Göttingen, Bonn, Toronto, Seattle.; 2001.

8. Snaith R., Zigmond AS. Hospital Anxiety Depression Scale. Deutsche Version. Bern: Huber; 2011.

**Figure S1.** Flowchart of participants and non-participants at baseline and follow-up.


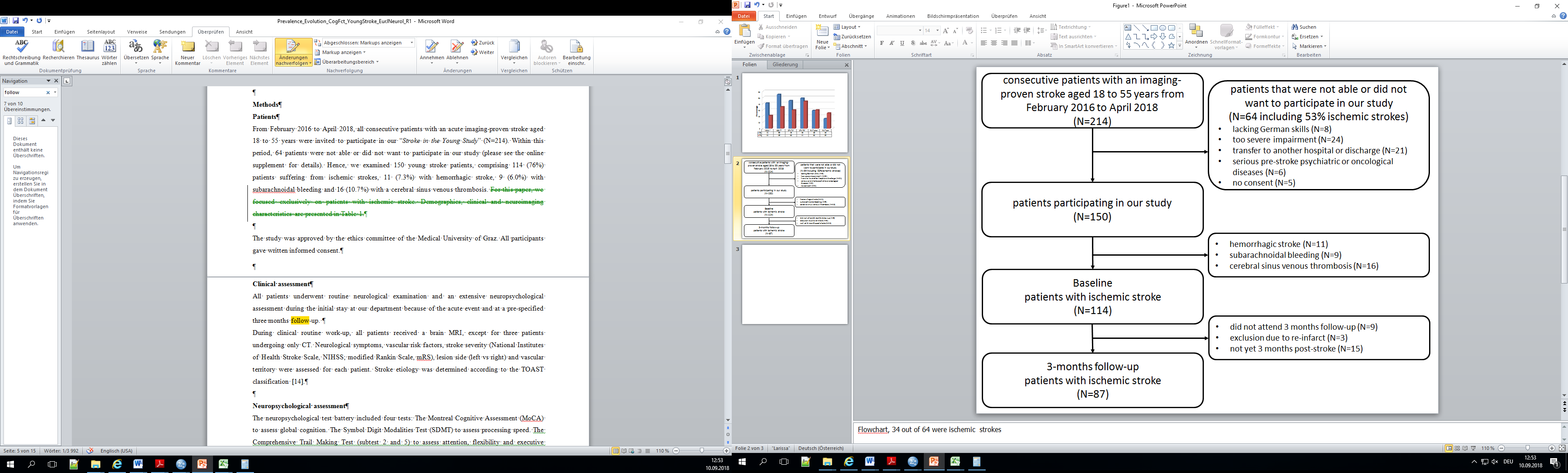


**Table S1.** Prevalence of clinical deficits at the three months follow-up (N=87). Motor and sensory impairment, aphasia and visual impairment according to NIHSS. Prevalence is indicated as number of patients N (%).

| **Characteristics at the three months follow-up** |  |
| --- | --- |
| NIHSS (Median, IQR) | 0 (1) |
| Visual impairment | 5 (5.7) |
| Motor impairment | 11 (12.6) |
| Sensory impairment | 15 (17.2) |
| Aphasia | 1 (1.1) |
| Dysarthria | 8 (9.2) |
